# Value of clinical tests in diagnosing anterior cruciate ligament injuries: A systematic review and meta-analysis

**DOI:** 10.1097/MD.0000000000029263

**Published:** 2022-08-05

**Authors:** Zhihao Huang, Zhihao Liu, Changfeng Fan, Miao Zou, Jiyan Chen

**Affiliations:** a Department of Physical Education, School of Fundamental Sciences, Shandong Institute of Petroleum and Chemical Technology, Dongying, China; b Department of Arts and Design, School of Education and Arts, Shandong Institute of Petroleum and Chemical Technology, Dongying, China; c Department of Mechanical Engineering, School of Mechanical and Control Engineering, Shandong Institute of Petroleum and Chemical Technology, Dongying, China.

**Keywords:** anterior cruciate ligament injuries, anterior drawer test, lachman test, lever sign test, meta-analysis, pivot shift test

## Abstract

**Objectives:**

This study compared 4 clinical tests with reference to magnetic resonance imaging and arthroscopic visualization to comprehensively evaluate their diagnostic value for anterior cruciate ligament injuries.

**Methods:**

We systematically searched 10 electronic databases from January 1, 2010, to May 1, 2021. Two reviewers collected data in accordance with the Preferred Reporting Item for Systematic Reviews and Meta-Analyses 2020 guidelines. The quality of each study was assessed using the Quality Assessment of Diagnostic Accuracy Studies 2 tool. A meta-analysis was performed using Meta-Disc version 1.4 and Stata SE version 15.0.

**Results:**

Eighteen articles involving 2031 participants were included. The results of the meta-analysis showed that for the Lachman test, the pooled sensitivity, specificity, positive likelihood ratio, negative likelihood ratio, diagnosis odds ratio, area under the curve (AUC) of summary receiver operating characteristic (SROC), and Q* were 0.76 (95% CI, 0.73–0.78), 0.89 (95% CI, 0.87–0.91), 5.65 (95% CI, 4.05–7.86), 0.28 (95% CI, 0.23–0.36), 22.95 (95% CI, 14.34–36.72), 0.88, and 0.81, respectively. For the anterior drawer test, the pooled sensitivity, specificity, positive likelihood ratio, negative likelihood ratio, diagnosis odds ratio, AUC of SROC, and Q* were 0.64 (95% CI, 0.61–0.68), 0.87 (95% CI, 0.84–0.90), 3.57 (95% CI, 2.13–5.96), 0.44 (95% CI, 0.32–0.59), 8.77 (95% CI, 4.11–18.74), 0.85, and 0.78, respectively. For the pivot shift test, the pooled sensitivity, specificity, positive likelihood ratio, negative likelihood ratio, diagnosis odds ratio, AUC of SROC, and Q* were 0.59 (95% CI, 0.56–0.62), 0.97 (95% CI, 0.95–0.98), 13.99 (95% CI, 9.96–19.64), 0.44 (95% CI, 0.35–0.55), 29.46 (95% CI, 15.60–55.67), 0.98, and 0.94, respectively. For the lever sign test, the pooled sensitivity, specificity, positive likelihood ratio, negative likelihood ratio, diagnosis odds ratio, AUC of SROC, and Q* were 0.79 (95% CI, 0.75–0.83), 0.92 (95% CI, 0.87–0.95), 9.56 (95% CI, 2.76–33.17), 0.23 (95% CI, 0.12–0.46), 47.38 (95% CI, 8.68–258.70), 0.94, and 0.87, respectively.

**Conclusions:**

Existing evidence shows that these clinical tests have high diagnostic efficacy for anterior cruciate ligament injuries, and that every test has its own advantages and disadvantages. However, the above results should be validated through additional studies, considering the limited quality and quantity of our sample.

## 1. Introduction

Clinically, anterior cruciate ligament (ACL) injuries are common athletic injuries that may compromise the stability of the knee joint. These injuries are usually caused by direct or indirect trauma of the knee joint. Along with economic development, the awareness of physical exercise is increasing. However, the number of patients with ACL injuries is also increasing. In the United States, approximately 35,000 procedures are performed annually for ACL reconstruction.^[1]^ Globally, the number of these procedures exceeds one million per year.^[2]^ Clinically, accurate early diagnosis of ACL injuries is important for selecting an appropriate treatment regimen and improving prognosis. Currently, clinical tests—the Lachman test (LT), anterior drawer test (ADT), pivot shift test (PST), lever sign test (LST)—and imaging procedures, such as radiography, ultrasound, computed tomography, magnetic resonance imaging (MRI), and arthroscopic visualization, are the most common methods for diagnosing ACL injuries. Radiography, ultrasound, and computed tomography are not precise in determining the location of injury and measurement. Moreover, for relevant operations, the requirements for operator’s experience are relatively high. MRI is expensive, with a possibility of measurement errors. Although invasive, arthroscopic visualization is the reference standard for the diagnosis of ACL injuries. Therefore, clinical testing is crucial. This study collected published articles on the 4 clinical tests (LT, ADT, PST, and LST) for the diagnosis of ACL injuries, in both English and Chinese worldwide. A meta-analysis was conducted to evaluate the value of the clinical tests for the diagnosis of ACL injuries to provide a reference for the early clinical diagnosis of ACL injuries.

## 2. Methods

This systematic review was conducted in accordance with Preferred Reporting Item for Systematic Reviews and Meta-Analyses (PRISMA) 2020 guidelines^[3]^ and was prospectively registered in International Prospective Register of Systematic Reviews (PROSPERO) (registration number: CRD42021256253).

The PubMed, Cochrane Library, Embase, Web of Science, CNKI, Wangfang Data, VIP, CBM, Chinese Clinical Trial Registry, and the ClinicalTrials.gov electronic databases were searched to collect relevant studies from January 1, 2010, to May 1, 2021. Furthermore, reference lists of the included studies were reviewed to supplement the relevant data.

### 2.1. Inclusion and exclusion criteria

#### 2.1.1. Inclusion criteria.

We included articles on the clinical tests for the diagnosis of ACL injuries in English or Chinese that have been published to date in which all the participants received tests as a reference standard, and the test results were explicitly diagnosed. There were no limitations regarding sex and age of the participants.

#### 2.1.2. Exclusion criteria.

For repeated articles, only the latest and most complete data were included. Additionally, the following articles were excluded: fundamental studies such as animal experiments, systematic reviews, conference papers, abstracts, lectures, and case reports; studies with unclear measurements, inappropriate statistical methods, or insufficiently described important outcome indicators; and literature with the results that cannot be extracted directly or indirectly.

### 2.2. Data collection

All of the retrieved articles were imported into the NoteExpress version 3.4 to find duplicate articles automatically. The remaining articles were screened primarily by reading the abstracts. Fully relevant texts were downloaded, and articles that met the relevant requirements, according to the abovementioned inclusion and exclusion criteria, were selected. Article screening, data extraction, and cross-checks were independently conducted by 2 reviewers. Differences, if any, were resolved through discussion or negotiation with a third reviewers. The following information was extracted from each of the studies: title; first author; journal of publication; baseline characteristics and diagnostic information of the participants; key elements of risk of bias assessment; and outcome indicators, including the values of true positive, false positive, false negative, and true negative, which were calculated or acquired directly.

### 2.3. Quality assessment

The risk of bias for the included studies was assessed using the Review Manager version 5.3, based on the Quality Assessment Tool of Diagnostic Accuracy Studies 2 (QUADAS-2).^[4]^ The results were further cross-checked independently by the 2 reviewers. Differences, if any, were resolved through discussion. Each item was regarded as “yes” (low bias or good suitability), “no” (high bias or poor suitability), or “unclear” (lack of relevant information or uncertainty regarding the bias).

### 2.4. Data analysis

Meta-Disc version 1.4 and Stata SE version 15.0 were used for the meta-analysis. The presence of a threshold effect was further tested using Spearman correlation analysis. A significant positive correlation between sensitivity and (1—specificity) indicated the presence of a threshold effect. Statistical heterogeneity among the studies was analyzed using the chi-squared test, and the magnitude of heterogeneity was determined based on *I^2^* values. In the case of statistical heterogeneity among the studies (*I^2^ >* 50%), a random-effects model was used for the pooled analysis after excluding significant clinical heterogeneity through meta-regression or subgroup analysis; otherwise, a fixed-effects model was used for the pooled analysis. Based on the corresponding model, the pooled sensitivity (Sen), specificity (Spe), positive likelihood ratio (+LR), negative likelihood ratio (−LR), and diagnostic odds ratio (DOR) of the included studies were calculated. The summary receiver operating characteristic (SROC) was further plotted, and the area under the curve (AUC) and Q* were calculated. The included studies were then excluded individually for the sensitivity analysis. If the results of the meta-analysis differed from the results of previous studies, the stability of the included studies was good; otherwise, the stability of the included studies was poor. In conclusion, Deek funnel plot was used to examine publication bias, with *P* > .05 indicating no publication bias for the included studies; otherwise, there was a publication bias.

## 3. Results

### 3.1. Literature search and characteristics of the included studies

**Figure 1.**
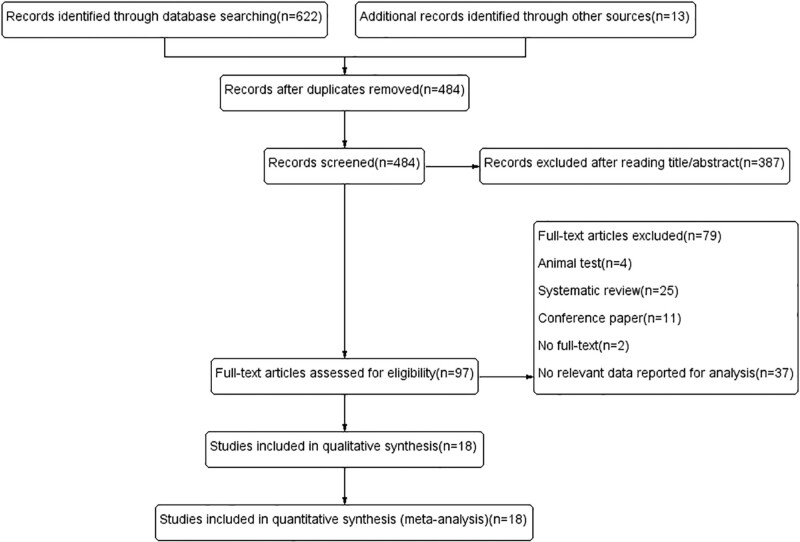
Flow diagram of the literature search and selection processes.

Following the search strategy, the initial search identified 635 articles. After elimination of duplicates, 484 articles remained. Then, 387 articles were further excluded after carefully reading the title and abstract. Next, 79 articles were excluded after reading the full text due to the following reasons: animal experiments (n = 4), systematic reviews (n = 25), conference papers (n = 11), lack of full text (n = 2), and lack of data reported for analysis (n = 37). In conclusion, 18 articles were included in the quantitative synthesis (meta-analysis).^[5–22]^ Figure 1, Supplemental Method 1, http://links.lww.com/MD/G869 presents detailed information on the flowchart regarding the search and selection of the literature.

Table 1 further summarizes the included studies, which included 2031 participants, including 25 LT studies reporting, 14 ADT studies reporting, 15 PST studies reporting, and 7 LST studies reporting.

**Table 1:** Characteristics of included studies

Study name	Country	Type of study	Sample size (male/female)	Age mean ± SD or (range)	Reported outcome
Han et al (2010)^[5]^	China	Prospective	80 (53/27)	28 (16–55)	LT; ADT; PST
Mulligan et al (2011)^[6]^	United states	Prospective	52 (31/21)	34.3 ± 12.0 (16–57)	LT
Zhao et al (2013)^[7]^	China	Retrospective	76 (46/30)	22.9	ADT
Mulligan et al (2015)^[8]^	United states	Prospective	45 (21/24)	40.7 ± 14.0 (20–64)	LT
Tanaka et al (2017)^[9]^	Japan	Retrospective	48 (N/A)	N/A	LT
Mulligan et al (2017)^[10]^	United states	Prospective	60 (38/22)	42.0 ± 13.4 (18–65)	LT; LST
Massey et al (2017)^[11]^	United states	Prospective	91 (61/30)	28.0 ± 11.0	LT; ADT; PST; LST
Wu et al (2017)^[12]^	China	Retrospective	100 (67/33)	34.9 (18–61)	LT; ADT; PST; LST
Cai et al (2017)^[13]^	China	Retrospective	210 (149/61)	31.2	LT; ADT
Kiyak et al (2018)^[14]^	Turkey	Prospective	62 (N/A)	N/A	LT; ADT; PST
Décary et al (2018)^[15]^	Canada	Prospective	279 (118/161)	49.1 ± 15.8	LT; PST
Lichtenberg et al (2018)^[16]^	Netherlands	Prospective	94 (57/37)	34.0 ± 15.0	LT; ADT; PST; LST
Krakowski et al (2019)^[17]^	Poland	Prospective	96 (49/47)	45.0 ± 16.0	LT; ADT; PST; LST
Gürpinar et al (2019)^[18]^	Turkey	Prospective	78 (69/9)	26.2 ± 6.4 (17–44)	LT; ADT; PST; LST
Blanke et al (2020)^[19]^	Germany	Retrospective	100 (62/38)	35.9 ± 16.8	LT; PST
Murgier et al (2020)^[20]^	France	Prospective	130 (89/41)	27.2 ± 8.3 (21–31)	PST
Feng (2020)^[21]^	China	Retrospective	30 (25/5)	29.3 ± 6.1	LT
Zhao et al (2021)^[22]^	China	Retrospective	400 (296/104)	28.7 ± 7.0	LT; ADT; PST

### 3.2. Risk of bias assessment in the included studies

**Figure 2.**
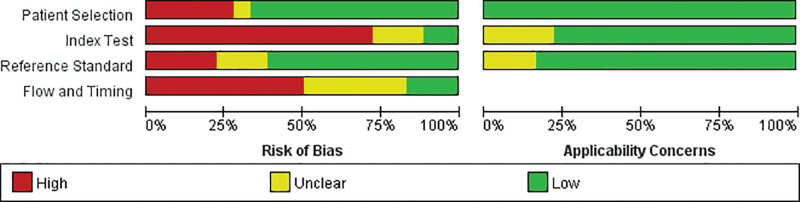
Risk of bias and applicability concerns graph.

**Figure 3.**
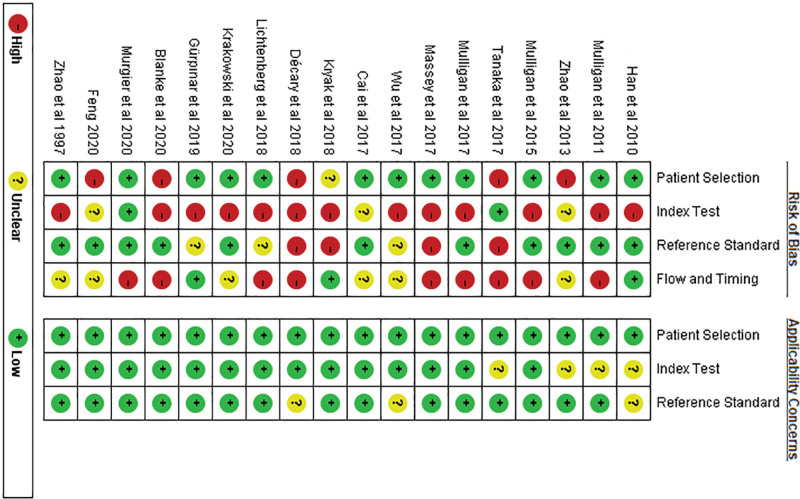
Risk of bias and applicability concerns summary.

The results of the QUADAS-2 evaluation of the quality of the included articles showed that the risk assessment in 4 aspects—patient selection, index test, reference standard, and flow and timing—was relatively unsatisfactory. The risk bias was relatively high, especially in terms of the index test and flow and timing. As for the index test, 14 articles either disregarded thresholds or the thresholds were not prespecified. In terms of flow and timing, for 9 of the articles, some cases were not included in the relevant analysis. This resulted in bias in the articles included in this study (Figs. 2 and 3; Supplemental Table 1, http://links.lww.com/MD/G869).

### 3.3. Meta-analysis of LT

Sixteen articles, with 25 studies on 2550 participants, were included in the meta-analysis (Table 1; Supplemental Table 2, http://links.lww.com/MD/G869).

#### 3.3.1. Heterogeneity test.

**Figure 4.**
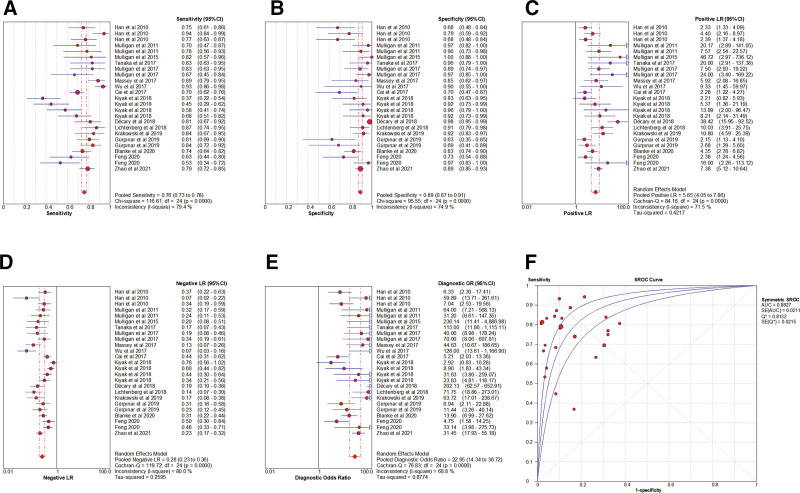
Forest plot for pooled effect sizes of LT for the diagnosis of ACL injuries. The subgraph of (A–F) refers to Sen, Spe, +LR, −LR, DOR, AUC, and Q*, respectively. ACL = anterior cruciate ligament, AUC = area under the curve, DOR = diagnostic odds ratio, +LR = positive likelihood ratio, −LR = negative likelihood ratio, LT = Lachman test.

The presence of the threshold effect was investigated by calculating the Spearman correlation coefficient between the Sen logarithm and the (1−Spe) logarithm. The correlation coefficient value of LT was 0.123 (*P* = .558), indicating that there was no threshold effect in this study. The heterogeneity test results showed that the heterogeneity of Sen (*χ^2^*= 116.61, *P* < .001, *I^2^*= 79.4%), Spe (χ*^2^*= 95.55, *P* < .001, *I^2^*= 74.9%), +LR (Cochran-Q = 84.16, *P* < .001, *I^2^*= 71.5%), −LR (Cochran-Q = 119.72, *P* < .001, *I^2^*= 80.0%), and DOR (Cochran-Q = 76.83, *P* < .001, *I^2^*= 68.8%) among the studies was large (Fig. 4). The cause of heterogeneity was not found through meta-regression or subgroup analysis. Therefore, the effect sizes were pooled using a random-effects model.

#### 3.3.2. The results of the meta-analysis.

The effect sizes of Sen_(pooled)_, Spe_(pooled)_, +LR_(pooled)_, −LR_(pooled)_, DOR_(pooled)_, AUC, and Q* of LT were 0.76 (95% CI, 0.73–0.78), 0.89 (95% CI, 0.87–0.91), 5.65 (95% CI, 4.05–7.86), 0.28 (95% CI, 0.23–0.36), 22.95 (95% CI, 14.34–36.72), 0.88 and 0.81, respectively (Fig. 4).

#### 3.3.3. Sensitivity analysis.

**Figure 5.**
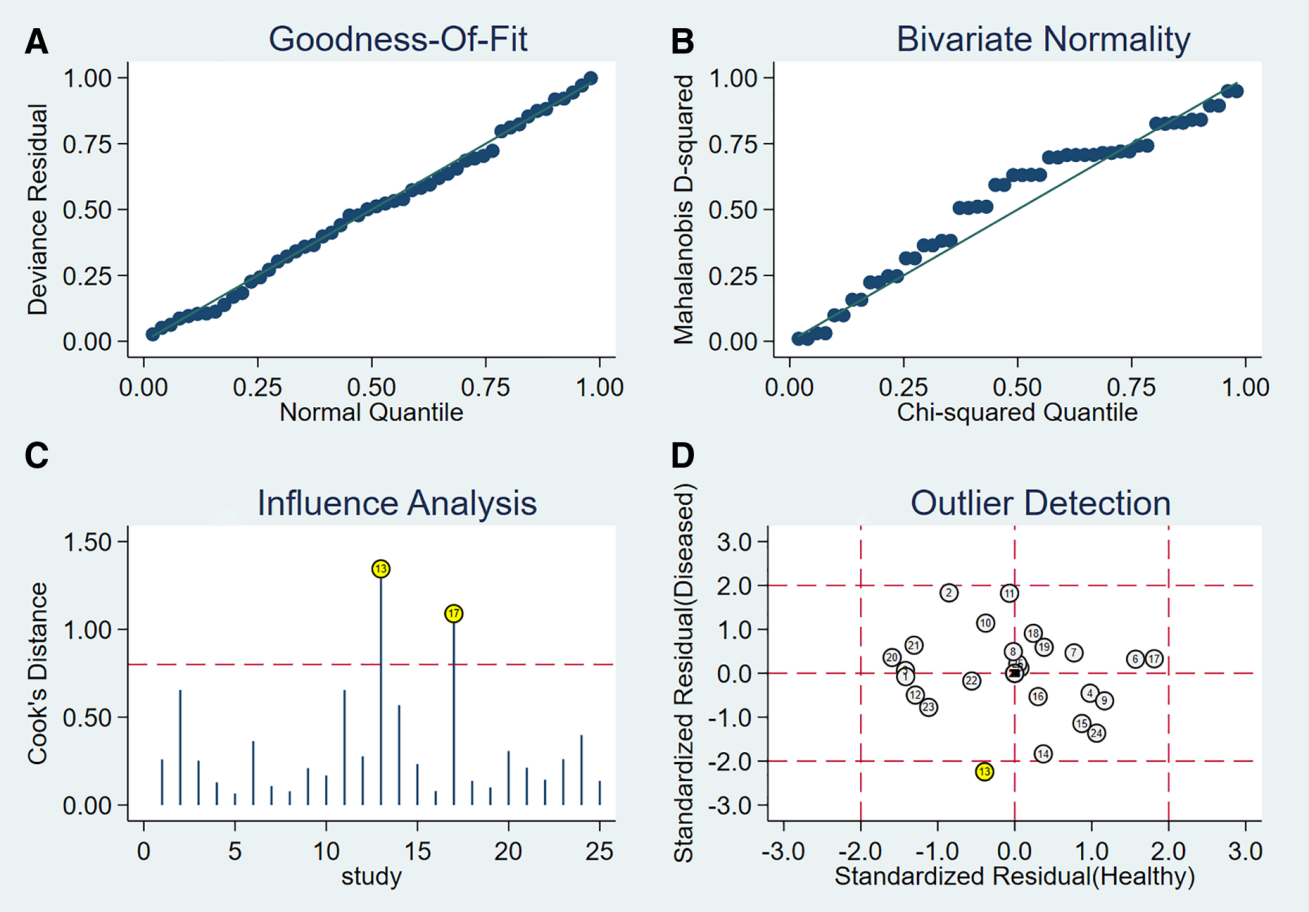
The sensitivity analysis of LT. LT = Lachman test.

A sensitivity analysis was conducted for the remaining studies after separately screening individual studies. The results showed that the effect of each eliminated study on the pooled effect size was relatively small, indicating that the results of this study were robust and the confidence level of the analysis results was high (Fig. 5).

#### 3.3.4. Analysis of the publication bias.

**Figure 6.**
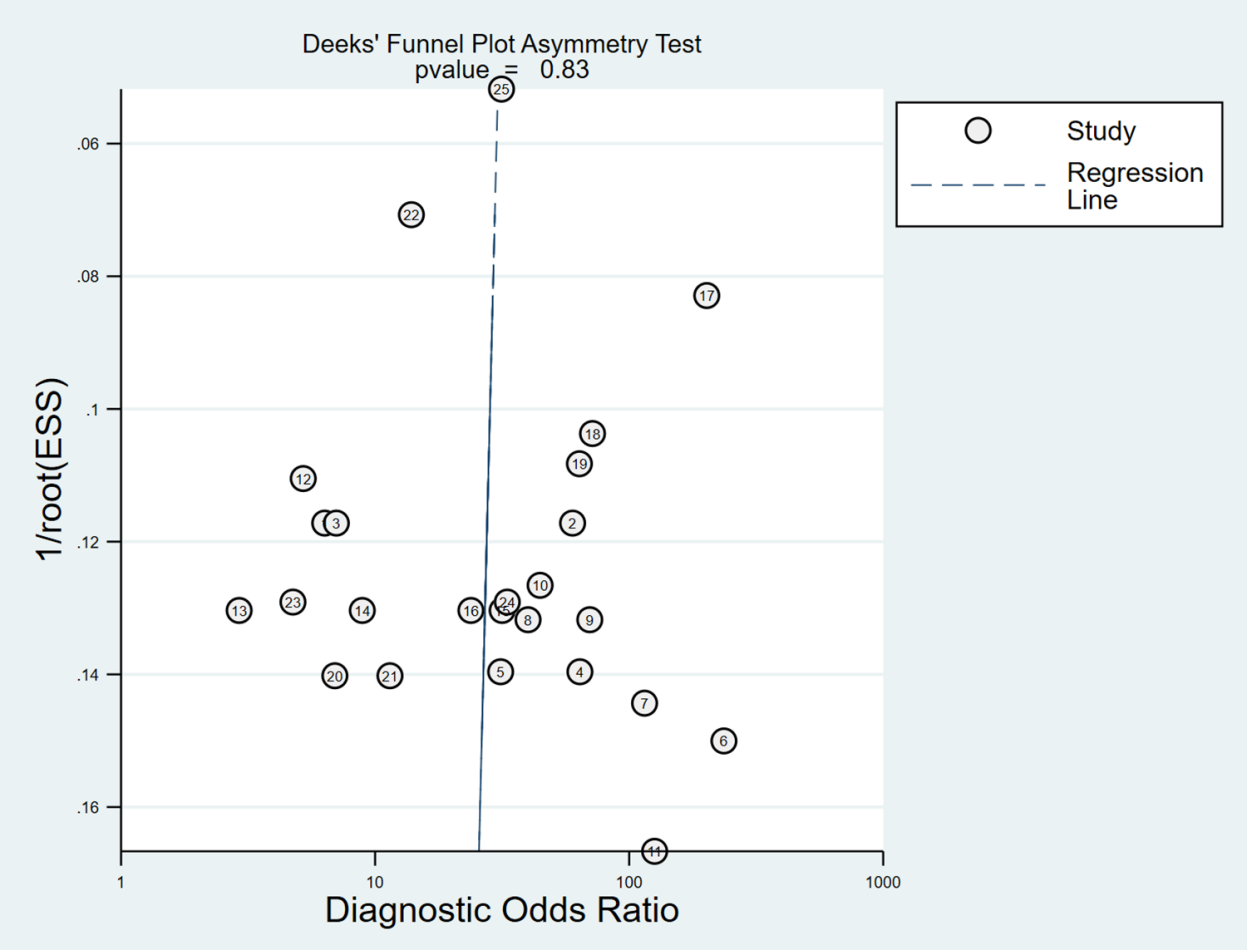
Funnel plot of LT for the diagnosis of ACL injuries. ACL = anterior cruciate ligament, LT = Lachman test.

Regarding the included studies concerning LT for the diagnosis of ACL injuries, a Deek’s funnel plot was given, with the inverse of the square root of the effective sample size (1/ESS1/2) as the vertical coordinate, and DOR as the abscissa coordinate.^[23]^ The result showed that there was no publication bias for LT (*P* = .83) (Fig. 6).

### 3.4. Meta-analysis of ADT

Ten articles, with 14 studies on 1548 participants, were included in the meta-analysis (Table 1; Supplemental Table 3, http://links.lww.com/MD/G869).

#### 3.4.1. Heterogeneity test.

**Figure 7.**
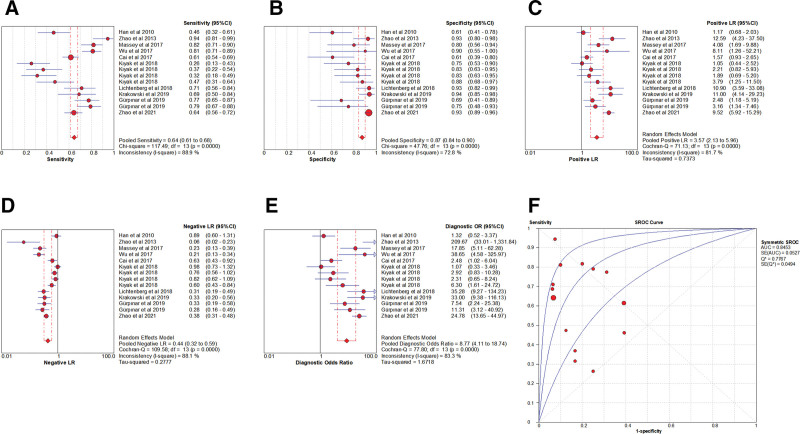
Forest plot for pooled effect sizes of ADT for the diagnosis of ACL injuries. The subgraph of (A–F) refer to Sen, Spe, +LR, −LR, DOR, AUC, and Q*, respectively. ACL = anterior cruciate ligament, AUC = area under the curve, ADT = anterior drawer test, DOR = diagnostic odds ratio, +LR = positive likelihood ratio, −LR = negative likelihood ratio.

The presence of the threshold effect was ascertained by calculating the Spearman correlation coefficient between the Sen logarithm and the (1−Spe) logarithm. The correlation coefficient value of ADT was −0.264 (*P* = .361), indicating that there was no threshold effect in this study. The heterogeneity test results showed that the heterogeneity of Sen (*χ^2^*= 117.49, *P* < .001, *I^2^* = 88.9%), Spe (*χ^2^*= 47.76, *P* < .001, *I^2^* = 72.8%), +LR (Cochran-Q = 71.13, *P* < .001, *I^2^*= 81.7%), −LR (Cochran’s Q = 109.58, *P* < .001, *I^2^*= 88.1%), and DOR (Cochran-Q = 77.80, *P* < .001, *I^2^*= 83.3%) among the studies was large (Fig. 7). The cause of heterogeneity was not found through meta-regression or subgroup analysis; therefore, the effect sizes were pooled using a random-effects model.

#### 3.4.2. The results of the meta-analysis.

The effect sizes of Sen_(pooled)_, Spe_(pooled)_, +LR_(pooled)_, −LR_(pooled)_, DOR_(pooled)_, AUC, and Q* of LT were 0.64 (95% CI, 0.61–0.68), 0.87 (95% CI, 0.84–0.90), 3.57 (95% CI, 2.13–5.96), 0.44 (95% CI, 0.32–0.59), 8.77 (95% CI, 4.11–18.74), 0.85, and 0.78, respectively (Fig. 7).

#### 3.4.3. Sensitivity analysis.

**Figure 8.**
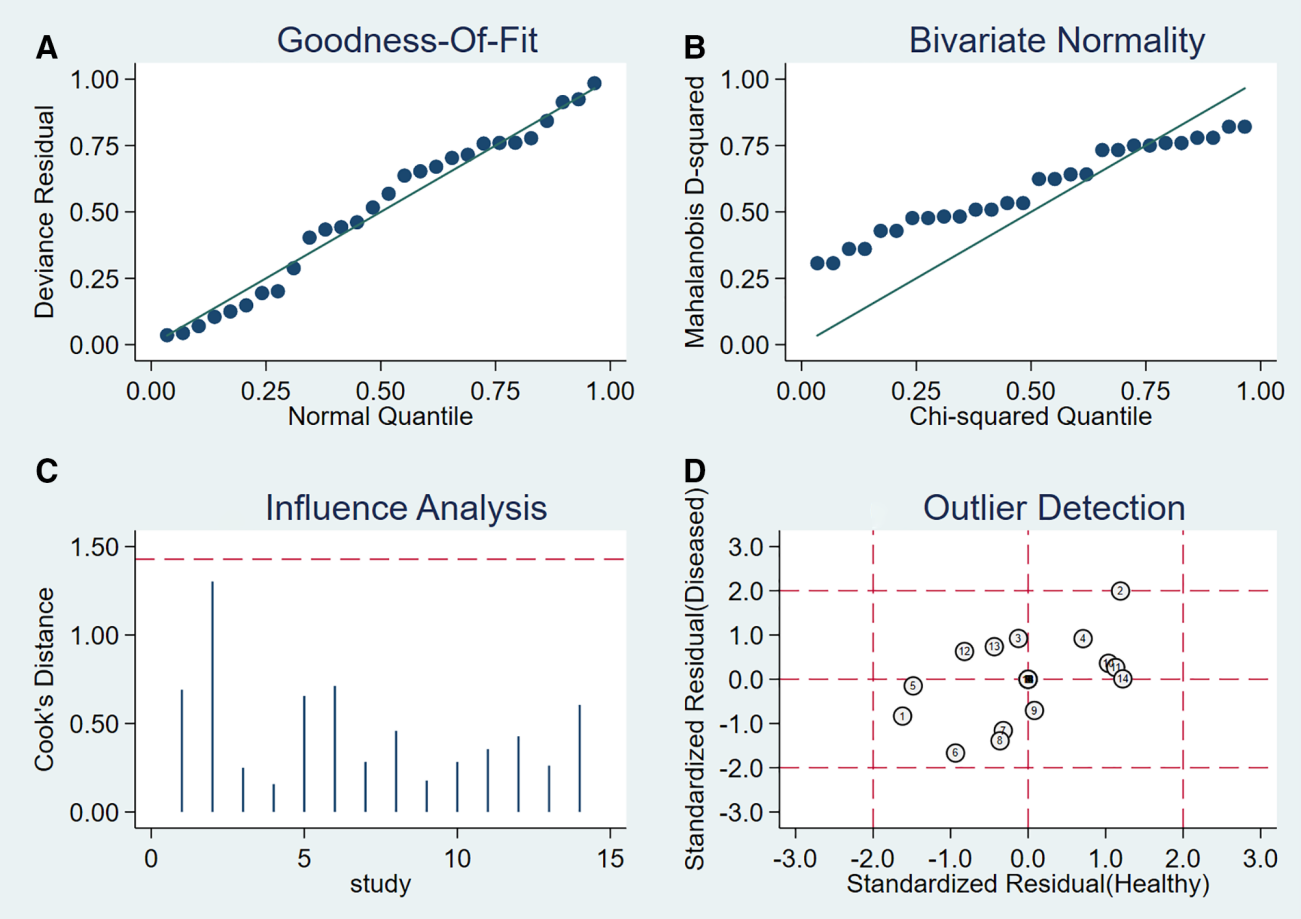
The sensitivity analysis of ADT. ADT = anterior drawer test.

A sensitivity analysis was conducted for the remaining studies after separately screening individual studies. The results showed that the effect of each eliminated study on the pooled effect size were relatively small, indicating that the results of this study were robust and that the confidence level of the analysis results was high (Fig. 8).

#### 3.4.4. Analysis of the publication bias.

**Figure 9.**
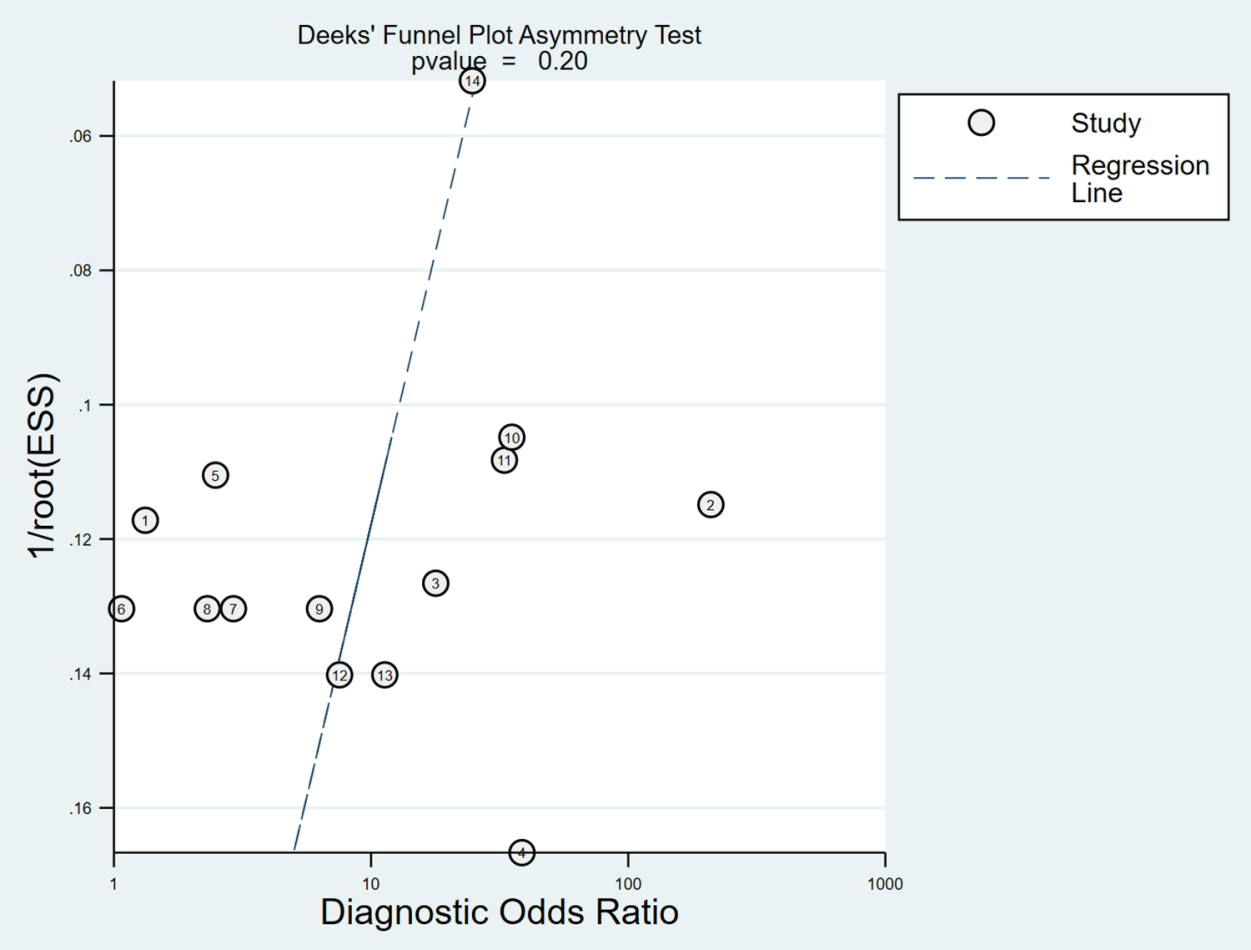
Funnel plot of ADT for the diagnosis of ACL injuries. ACL = anterior cruciate ligament.

Regarding the included studies concerning ADT for the diagnosis of ACL injuries, a Deek’s funnel plot was given, with the inverse of the square root of the effective sample size (1/ESS1/2) as the vertical coordinate, and DOR as the abscissa coordinate.^[23]^ The result showed that there was no publication bias for ADT (*P* = .20) (Fig. 9).

### 3.5. Meta-analysis of PST

Eleven articles, with 15 studies on 1853 participants, were included in the meta-analysis (Table 1; Supplemental Table 4, http://links.lww.com/MD/G869).

#### 3.5.1 . Heterogeneity test.

**Figure 10.**
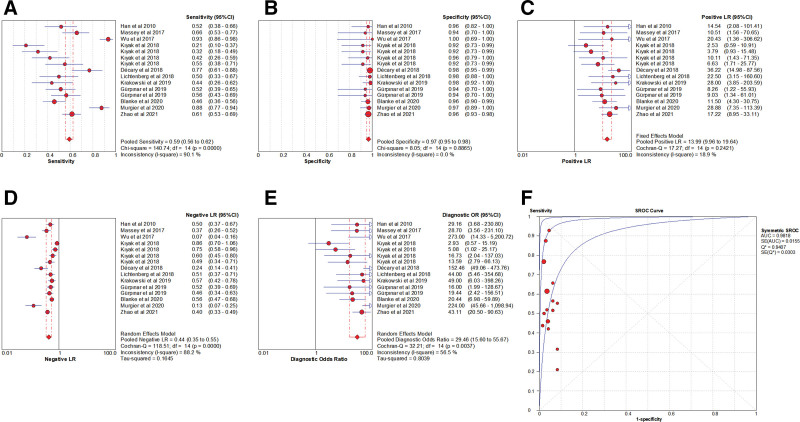
Forest plot for pooled effect sizes of PST for the diagnosis of ACL injuries. The subgraph of (A–F) refers to Sen, Spe, +LR, −LR, DOR, AUC, and Q*, respectively. +LR = positive likelihood ratio, −LR = negative likelihood ratio, ACL = anterior cruciate ligament, AUC = area under the curve, DOR = diagnostic odds ratio, PST = pivot shift test.

The presence of the threshold effect was ascertained by calculating the Spearman correlation coefficient between the Sen logarithm and the (1−Spe) logarithm. The correlation coefficient value of PST was −0.254 (*P* = .361), indicating that there was no threshold effect in this study. The heterogeneity test results showed that the heterogeneity of Sen (*χ^2^* = 140.74, *P* < .001, *I^2^* = 90.1%), −LR (Cochran-Q = 118.51, *P* < .001, *I^2^* = 88.2%), and DOR (Cochran-Q = 32.21, *P* < .001, *I^2^* = 56.5%) among the studies was large, and the cause of heterogeneity was not found through meta-regression or subgroup analysis. Therefore, the effect sizes were pooled using a random-effects model. In contrast, the heterogeneity of Spe (*χ^2^* = 8.05, *P* = .887, *I^2^* = 0.0%) and +LR (Cochran-Q = 17.27, *P* = .242, *I^2^* = 18.9%) among the studies was small; hence, the effect sizes were pooled using a fixed-effect model (Fig. 10).

#### 3.5.2. The results of the meta-analysis.

The effect sizes of Sen_(pooled)_, Spe_(pooled)_, +LR_(pooled)_, −LR_(pooled)_, DOR_(pooled)_, AUC, and Q* of LT were 0.59 (95% CI, 0.56–0.62), 0.97 (95% CI, 0.95–0.98), 13.99 (95% CI, 9.96–19.64), 0.44 (95% CI, 0.35–0.55), 29.46 (95% CI, 15.60–55.67), 0.98, and 0.94, respectively (Fig. 10).

#### 3.5.3. Sensitivity analysis.

**Figure 11.**
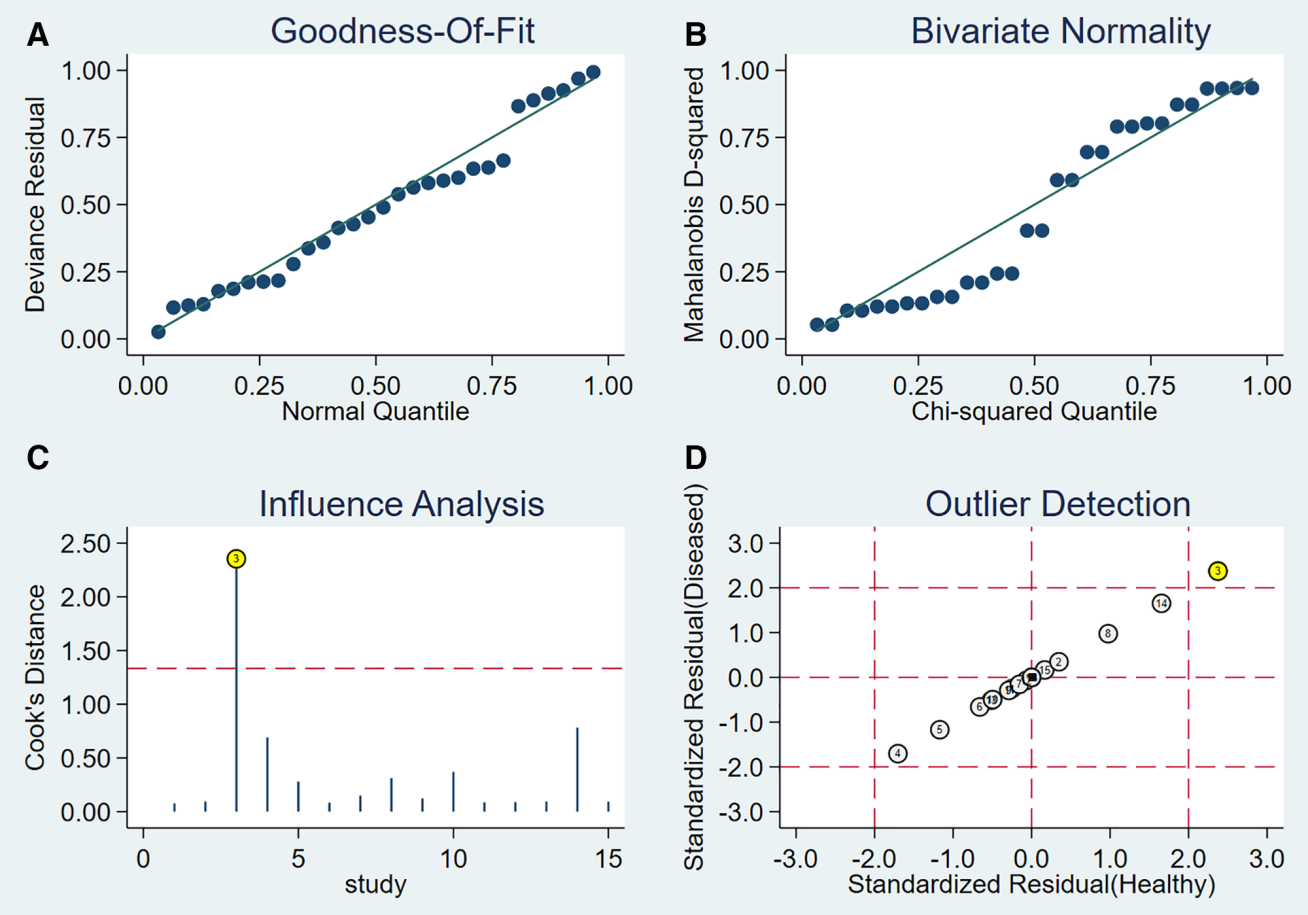
The sensitivity analysis of PST. PST = pivot shift test.

A sensitivity analysis was conducted for the remaining studies after separately screening individual studies. The results showed that the effect of each eliminated study on the pooled effect size were relatively small, indicating that the results of this study were robust and that the confidence level of the analysis results was high (Fig. 11).

#### 3.5.4. Analysis of the publication bias.

**Figure 12.**
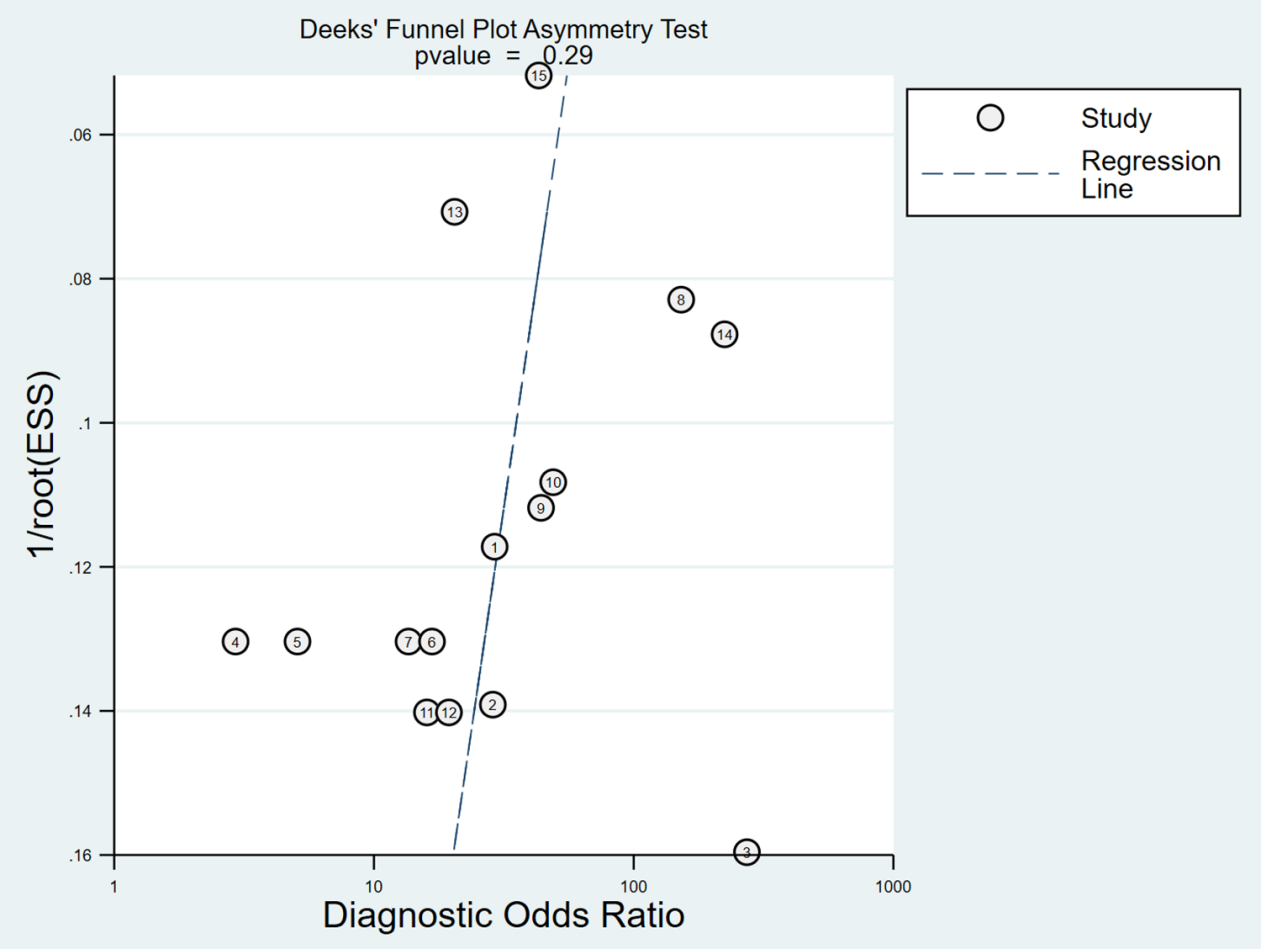
Funnel plot of PST for the diagnosis of ACL injuries. ACL = anterior cruciate ligament, PST = pivot shift test.

Regarding the included studies concerning PST for the diagnosis of ACL injuries, a Deek funnel plot was given, with the inverse of the square root of the effective sample size (1/ESS1/2) as the vertical coordinate, and DOR as the abscissa coordinate.^[23]^ The result showed that there was no publication bias for PST (*P* = .29) (Fig. 12).

### 3.6. Meta-analysis of LST

Six articles, with 7 studies on 590 participants, were included in the meta-analysis (Table 1; Supplemental Table 5, http://links.lww.com/MD/G869).

#### 3.6.1. Heterogeneity test.

**Figure 13.**
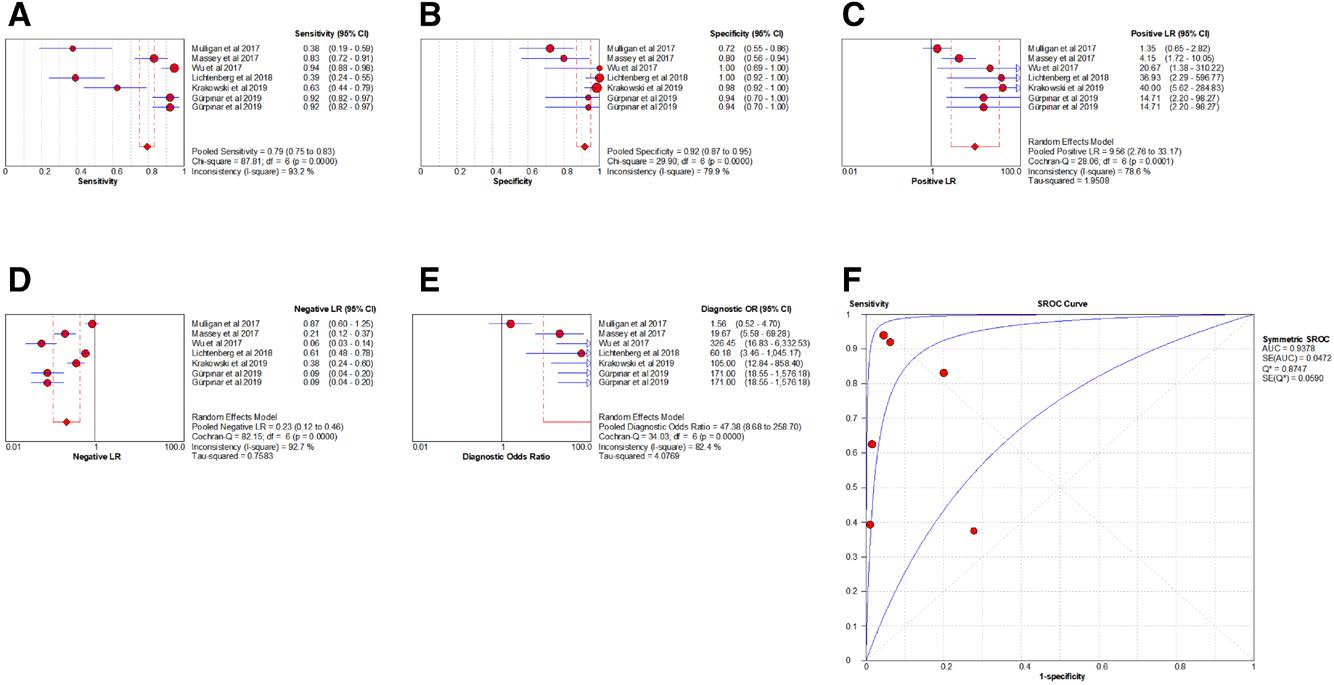
Forest plot for pooled effect sizes of LST for the diagnosis of ACL injuries. The subgraph of (A–F) refers to Sen, Spe, +LR, −LR, DOR, AUC, and Q*, respectively. +LR = positive likelihood ratio, −LR = negative likelihood ratio, ACL = anterior cruciate ligament, AUC = area under the curve, DOR = diagnostic odds ratio, LST = lever sign test.

The presence of the threshold effect was ascertained by calculating the Spearman correlation coefficient between the Sen logarithm and the (1−Spe) logarithm. The correlation coefficient value of LST was −0.091 (*P* = .846), indicating that there was no threshold effect in this study. The heterogeneity test results showed that the heterogeneity of Sen (*χ^2^* = 87.81, *P* < .001, *I^2^* = 93.2%), Spe (*χ^2^* = 29.90, *P* < .001, *I^2^* = 79.9%), +LR (Cochran’s Q = 28.06, *P* < .001, *I^2^*= 78.6%), −LR (Cochran’s Q = 82.15, *P* < .001, *I^2^* = 92.7%), and DOR (Cochran’s Q = 34.03, *P* < .001, *I^2^* = 82.4%) among the studies was large (Fig. 13). The cause of heterogeneity was not found through meta-regression or subgroup analysis; therefore, the effect sizes were pooled using a random-effects model.

#### 3.6.2. The results of the meta-analysis.

The effect sizes of Sen_(pooled)_, Spe_(pooled)_, +LR_(pooled)_, −LR_(pooled)_, DOR_(pooled)_, AUC, and Q* of LT were 0.79 (95% CI, 0.75–0.83), 0.92 (95% CI, 0.87–0.95), 9.56 (95% CI, 2.76–33.17), 0.23 (95% CI, 0.12–0.46), 47.38 (95% CI, 8.68–258.70), 0.94, and 0.87, respectively (Fig. 13).

#### 3.6.3. Sensitivity analysis.

**Figure 14.**
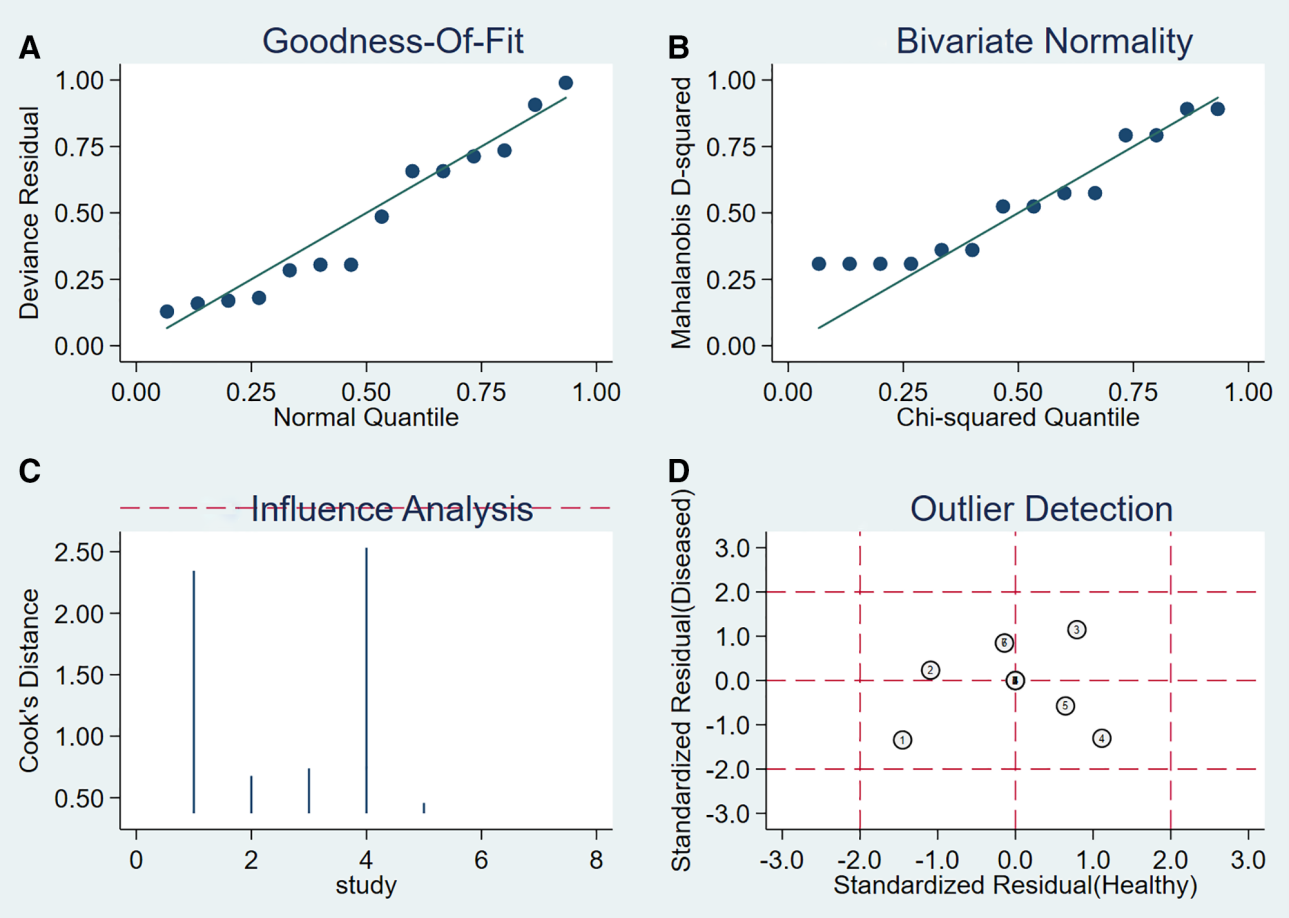
The sensitivity analysis of LST. LST = lever sign test.

A sensitivity analysis was conducted for the remaining studies after separately screening individual studies. The results showed that the effect of each eliminated study on the pooled effect size were relatively small, indicating that the results of this study were robust and that the confidence level of the analysis results was high (Fig. 14).

#### 3.6.4. Analysis of the publication bias.

**Figure 15.**
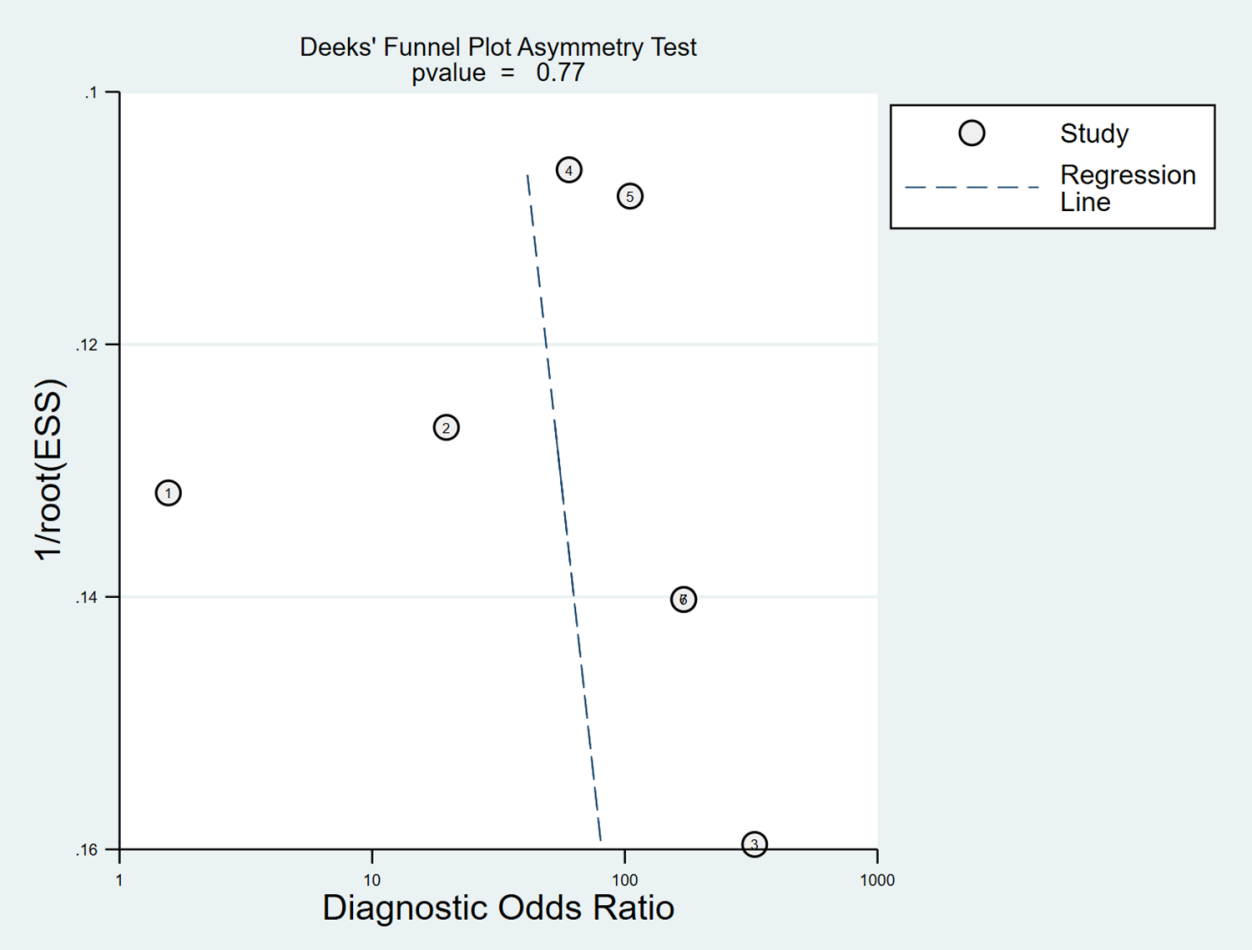
Funnel plot of LST for the diagnosis of ACL injuries. ACL = anterior cruciate ligament, LST = lever sign test.

Regarding the included studies concerning LST for the diagnosis of ACL injuries, a Deek funnel plot was given, with the inverse of the square root of the effective sample size (1/ESS1/2) as the vertical coordinate, and DOR as the abscissa coordinate.^[23]^ The result showed that there was no publication bias for LST (*P* = .77) (Fig. 15).

## 4. Discussion

The results of previous studies were combined quantitatively using a meta-analysis, in which the results of previous relevant independent studies were reviewed critically and combined statistically, and similar results were integrated quantitatively. Through a comprehensive evaluation of the inconsistency or contradiction of the study results, the sample size may be enlarged; the power of statistical tests may be advanced; and the shortcomings of previous studies may be simultaneously identified, thereby revealing the uncertainties of individual studies, and putting forward new topics and interests for relevant studies.

This study encompassed 18 articles, with arthroscopy, surgical exploration, and MRI as the reference standards for clinical tests in diagnosing ACL injuries. Our meta-analysis showed that the pooled sensitivity of LT, ADT, PST, and LST for diagnosing ACL injuries were 0.76, 0.64, 0.59, and 0.79, respectively, whereas the pooled specificity were 0.89, 0.87, 0.97, and 0.92, respectively. This suggests that the capability of the 4 clinical tests to diagnose ACL injuries was high. LRs are defined as the likelihood that a particular test result would be found in a patient with the target disorder, relative to the likelihood of the same test result occurring in a patient without the target disorder; the positive likelihood ratio—that is, the ratio of the true-positive rate to the false-positive rate for the screening results—indicates the ratio of probability of the screening test having a correct judgment for a positive result and its probability of wrong judgment for a positive result. A higher +LR indicates a greater probability that a positive test result is a true positive result. The negative likelihood ratio—that is, the ratio of the false-positive rate to the true-positive rate for the screening results—indicates the ratio of the probability of the screening test having a wrong judgment for a positive result to its probability of correct judgment for a positive result. A lower −LR indicates a greater probability that a negative test result is a true negative result.^[24]^ The pooled +LRs of LT, ADT, PST, and LST for diagnosing ACL injuries were 5.65, 3.57, 13.99, and 9.56, respectively, suggesting that when LT, PST, and LST diagnose ACL injuries as positive, the possibility of ACL injuries is high. The pooled −LRs of LT, ADT, PST, and LST for diagnosing ACL injuries were 0.28, 0.44, 0.44, and 0.23, respectively, indicating that when the 4 clinical tests are negative for the diagnosis of ACL injuries, ACL injuries are very likely to be excluded when there is a negative result on the 4 clinical tests. The DOR is the ratio of the odds of disease in positive tests relative to the odds of disease in negative tests. The value of DOR ranges from 0 to infinity, with higher values indicating better discriminatory test performance. A value of 1 means that a test does not discriminate between patients with and without the disorder. Values lower than 1 indicate improper test interpretation (more negative tests among the diseased).^[25]^ The DORs of LT, ADT, PST, and LST for the diagnosis of ACL injuries were 22.95, 8.77, 29.46, and 47.38, respectively, suggesting that the accuracy of the 4 clinical tests for the diagnosis of ACL injuries is high. The SROC considers both sensitivity and specificity, and comprehensively compares several clinical tests for importance on the basis of the AUC of the SROC. In terms of the AUC, the larger the value, the more important it is.^[26]^ The AUCs of LT, ADT, PST, and LST for diagnosing ACL injuries were 0.88, 0.85, 0.98, and 0.94, respectively; the Q* values of LT, ADT, PST, and LST for diagnosing ACL injuries were 0.81, 0.78, 0.94, and 0.87, respectively, which indicates that the 4 clinical tests have a high diagnostic efficiency for ACL injuries. ADT is a significant method for the clinical diagnosis of ACL injuries. However, despite its wide use in clinical practice, this method has some limitations. On one hand, acute patients often cannot cooperate effectively, owing to intra-articular hematoma and local pain in the affected limb; moreover, the knee joint cannot maintain flexion at 90°. On the other hand, when the knee joint is flexed at 90°, the meniscus attached to the medial tibia adheres to the convex surface of the medial femoral condyle at the posterior angle, inducing a “door stopper” effect and preventing the tibia from moving forward, which results in a false-positive result.^[27]^ Furthermore, when the posterior cruciate ligament relaxes or ruptures, the tibia may move forward, simply for the return of the femur from the place of subsidence to the medial starting position, which may cause misdiagnosis.^[12]^ Ostrowski et al^[28]^ reported that the overall sensitivity of ADT was only 20% (range, 18%–92%), while the specificity was 88% (range, 78%–98%). Benjaminse et al^[29]^ reported that ADT could yield good results in chronic patients, with a sensitivity of 0.92 (95% CI, 0.88–0.95) and specificity of 0.91 (95% CI, 0.87–0.94). LT can be considered as an ADT of 15° flexion. ACL injuries were determined mainly by observing the movement degree of tibia and femur on the anterior and posterior axes at 15° knee flexion.^[12]^ Therefore, LT can be used to examine patients with acute joint swelling, pain, and inability to flex the knee to 90°. When the knee joint flexes at 15°, the relatively flat joint of the femur no longer blocks the forward movement of the meniscus and tibia, thereby overcoming the disadvantages of ADT.^[30]^ However, when the posterior cruciate ligament relaxes or ruptures, misdiagnosis is also possible with LT.^[31]^ Rosenberg et al^[32]^ investigated the effect of clinical examinations on ACL tension and found that LT with the knee bent at 15° could produce the maximum tension in most ACL areas, while ADT with the knee bent at 90° could not produce the maximum tension in any part of the ACL. The principle of PST is based on imitation of the mechanism of ACL injuries. Therefore, PST is often affected by the patient’s muscle tension, protective response induced by pain, and range of motion, which significantly compromises the accuracy of the examination. However, under anesthesia, PST is relatively reliable.^[12]^ In fact, LST partially utilizes the lever principle first proposed by the ancient Greek scientist Archimedes in his “On the Equilibrium of Planes”; volume 1 of the work contains “the law of the lever,” which states that to balance the lever, the 2 torques acting on the lever (the product of the force and the moment arm) must be equal, that is, the power × power arm = resistance × resistance arm. In this process, the lever passing through the fulcrum may provide force conduction. One of the prerequisites for the principle and formula is the integrity of the lever. After ACL rupture, the downward pressure exerted on the thigh cannot move the weight of the leg and foot through the lever formed by the knee joint and the calf, as the continuous transmission of the lever force has been destroyed, and at this point, there will be a positive result of the lever test. The lever test can overcome the disadvantages of the above 3 tests: it does not require much experience for the examiner, the procedure is simple, and the patient’s pain is not increased. Lelli et al reported that the sensitivity of LST in the diagnosis of chronic complete ruptures of the ACL was close to 100%, especially in the diagnosis of acute and partial ruptures of the ACL. Moreover, the sensitivity was significantly higher than that of the other clinical tests.^[33]^

### 4.1. Limitations of this study

All of the articles included in this study were written in English and Chinese; therefore, there was a certain selection bias. Differences in the number of included patients, as well as their sex, age, and degree of ACL injuries, may have caused high heterogeneity. There were certain differences in the diagnostic criteria and reference standards between various studies. The differences in duration between clinical tests and reference standard tests may result in interpretation bias, which may have also affected the results of this study. By meta-regression, we did not find the source of the heterogeneity, which implies that the accuracy of the 4 clinical tests in the diagnosis of ACL injuries significantly depends on the skill and experience of the operators and the severity of the injuries.

## 5. Conclusion

In summary, the 4 clinical tests have a certain value in the diagnosis of ACL injuries. Moreover, each clinical test has both strengths and limitations. In clinical practice, the 4 clinical tests can be integrated to improve diagnostic performance. Considering the limitations in the number and quality of the included studies, relevant conclusions still need to be verified through more high-quality studies.

## Author contributions

Conceptualization: Zhihao Huang, Zhihao Liu, Changfeng Fan.

Formal analysis: Zhihao Huang, Zhihao Liu, Jiyan Chen.

Investigation: Zhihao Liu.

Methodology: Zhihao Huang, Miao Zou.

Project administration: Zhihao Huang.

Software: Zhihao Huang.

Supervision: Zhihao Huang, Zhihao Liu.

Visualization: Changfeng Fan.

Writing-original draft: Zhihao Huang.

Writing-review and editing: Zhihao Huang, Zhihao Liu, Changfeng Fan, Miao Zou, Jiyan Chen.

## Supplementary Material


