# Supplementary material for: Value of clinical tests in diagnosing anterior cruciate ligament injuries: A systematic review and meta-analysis
Source: Medicine (Baltimore). 2022 Aug 5;101(31):e29263. doi: 10.1097/MD.0000000000029263 (PMC9351841; doi:10.1097/MD.0000000000029263)
Supplement: Supplementary file 1 [file medi-101-e29263-s001.pdf]

Supplemental Digital Content

Supplemental Method 1 Electronic search strategy.

PubMed

#1 ((((((((((((((((((((((ACL injuries[Title/Abstract]) OR (ACL injury[Title/Abstract])) OR (A CL strain[Title/Abstract])) OR (ACL strains[Title/Abstract])) OR (ACL tear[Title/Abstract])) OR (ACL tears[Title/Abstract])) OR (ACL rupture[Title/Abstract])) OR (ACL ruptures[Title/ Abstract])) OR (Injuries,ACL[Title/Abstract])) OR (Injury,ACL[Title/Abstract])) OR (Strain,A CL[Title/Abstract])) OR (Strains,ACL[Title/Abstract])) OR (Tear,ACL[Title/Abstract])) OR (T ears,ACL[Title/Abstract])) OR (Rupture,ACL[Title/Abstract])) OR (Ruptures,ACL[Title/Abstra ct])) OR (Anterior Cruciate Ligament Injury[Title/Abstract])) OR (Anterior Cruciate Ligame nt Injuries[Title/Abstract])) OR (Anterior Cruciate Ligament Tear[Title/Abstract])) OR (Ante rior Cruciate Ligament Tears[Title/Abstract])) OR (Anterior Cruciate Ligament Strain[Title/ Abstract])) OR (Anterior Cruciate Ligament Strains[Title/Abstract])) OR (Anterior Cruciate Ligament Rupture[Title/Abstract])) OR (Anterior Cruciate Ligament Ruptures[Title/Abstract])

#2 (((((((lanchman test[Title/Abstract]) OR (LT[Title/Abstract])) OR (anterior drawer test[Tit le/Abstract])) OR (ADT[Title/Abstract])) OR (pivot shift test[Title/Abstract])) OR (PST[Title /Abstract])) OR (lever sign test[Title/Abstract])) OR (LST[Title/Abstract])

#3 sensitiv*[Title/Abstract] OR sensitivity and specificity[MeSH Terms] OR (predictive[Title/ Abstract] AND value*[Title/Abstract]) OR predictive value of tests[Title/Abstract] OR accura cy*[Title/Abstract]

#1 AND #2 AND #3

Note:We searched 58 articles while using PubMed as the filter. Searching date: January 1, 2010 to May 1, 2021.

Cochran Library

#1 (ACL Injuries):ab,ti,kw OR (ACL Injury):ab,ti,kw OR (ACL strain):ab,ti,kw OR (ACL s trains):ab,ti,kw OR (ACL Tears):ab,ti,kw OR (ACL Tear):ab,ti,kw OR (ACL Rupture):ab,ti,k w OR (ACL Ruptures):ab,ti,kw OR (Injuries, ACL):ab,ti,kw OR (Injury, ACL):ab,ti,kw OR (Strain, ACL):ab,ti,kw OR (Strains, ACL):ab,ti,kw OR (Tear, ACL):ab,ti,kw OR (Tears, A CL):ab,ti,kw OR (Rupture ,ACL):ab,ti,kw OR (Ruptures,ACL):ab,ti,kw OR (Anterior Crucia te Ligament Injury):ab,ti,kw OR (Anterior Cruciate Ligament Injuries):ab,ti,kw OR (Anterio r Cruciate Ligament Tear):ab,ti,kw OR (Anterior Cruciate Ligament Strain):ab,ti,kw OR (A nterior Cruciate Ligament Strains):ab,ti,kw OR (Anterior Cruciate Ligament Tears):ab,ti,kw OR (Anterior Cruciate Ligament Rupture):ab,ti,kw OR (Anterior Cruciate Ligament Rupture s):ab,ti,kw

#2 (Lachman test):ab,ti,kw OR (LT):ab,ti,kw OR (pivot shift test):ab,ti,kw OR (PST):ab,ti, kw (anterior drawer test):ab,ti,kw OR (ADT):ab,ti,kw

#3 (sensitiv):ab,ti,kw OR (sensitivity and specificity):ab,ti,kw OR (predictive):ab,ti,kw OR (predictive value of tests):ab,ti,kw OR (accuracy):ab,ti,kw

#1 AND #2 AND #3

Note:We searched 8 articles while using RefMan-(RIS) as the filter. Searching date: Januar y 1, 2010 to May 1, 2021.

Embase

#1 'ACL Injuries':ab,ti OR 'ACL Injury':ab,ti OR 'ACL strain':ab,ti OR 'ACL strains':ab,ti

OR 'ACL Tears':ab,ti OR 'ACL Tear':ab,ti OR 'ACL Rupture':ab,ti OR 'ACL Ruptures':ab,ti OR 'Injuries, ACL':ab,ti OR 'Injury, ACL':ab,ti OR 'Strain, ACL':ab,ti OR 'Strains, ACL':ab,ti OR 'Tear, ACL':ab,ti OR 'Tears, ACL':ab,ti OR 'Rupture ,ACL':ab,ti OR 'Ruptures,ACL':ab,ti OR 'Anterior Cruciate Ligament Injury':ab,ti OR 'Anterior Cruciate Ligament Injuries':ab,ti OR 'Anterior Cruciate Ligament Tear':ab,ti OR 'Anterior Cruciate Ligament Strain':ab,ti OR 'Anterior Cruciate Ligament Strains':ab,ti OR 'Anterior Cruciate Ligament Tears':ab,ti OR 'Anterior Cruciate Ligament Rupture':ab,ti OR 'Anterior Cruciate Ligament Ruptures':ab,ti

#2 'Lachman test':ab,ti OR 'LT':ab,ti OR 'pivot shift test':ab,ti 0R 'PST':ab,ti OR 'anterior drawer test':ab,ti OR 'ADT':ab,ti

#3 'sensitiv':ab,ti OR 'sensitivity and specificity':ab,ti OR 'predictive':ab,ti OR 'predictive value of tests':ab,ti OR 'accuracy':ab,ti

#1 AND #2 AND #3

Note:We searched 43 articles while using RefMan-(RIS) as the filter. Searching date: January 1, 2010 to May 1, 2021.

Web of Science

#1 TS=(ACL Injuries or ACL Injury or ACL strain or ACL strains or ACL Tears or ACL Tear or ACL Rupture or ACL Ruptures or Injuries, ACL or Injury, ACL or Strain, ACL or Strains, ACL or Tear, ACL or Tears, ACL or Rupture ,ACL or Ruptures,ACL or Anterior Cruciate Ligament Injury or Anterior Cruciate Ligament Injuries or Anterior Cruciate Ligament Tear or Anterior Cruciate Ligament Strain or Anterior Cruciate Ligament Strains or Anterior Cruciate Ligament Tears or Anterior Cruciate Ligament Rupture or Anterior Cruciate Ligament Ruptures)

#2 TS=(Lachman test or LT or pivot shift test or PST or anterior drawer test or ADT)

#3 TS=(sensitiv or sensitivity and specificity or predictive or predictive or predictive value of tests or accuracy)

#4 #1 and #2 and #3

Note:We searched 55 articles while using Web of Science as the filter. Searching date: January 1, 2010 to May 1, 2021.

CNKI

#1 前交叉韧带损伤 OR 前交叉韧带拉伤 OR 前交叉韧带撕裂 OR 前交叉韧带断裂 OR 前十字韧带损伤 OR 前十字韧带拉伤 OR 前十字韧带撕裂 OR 前十字韧带断裂

#2 拉赫曼试验 OR lachman 试验 OR 轴移试验 OR 前抽屉试验

#3 诊断

#4 #1 AND #2 AND #3

Note:We searched 51 articles while using NoteExpress as the filter. Searching date: January 1, 2010 to May 1, 2021.

Wangfang Data

主题：(前交叉韧带损伤 or 前交叉韧带拉伤 or 前交叉韧带撕裂 or 前交叉韧带断裂 or 前十字韧带损伤 or 前十字韧带拉伤 or 前十字韧带撕裂 or 前十字韧带断裂) and 主题：(拉赫曼试验 or lachman 试验 or 轴移试验 or 前抽屉试验) and 主题：(诊断)

Note:We searched 126 articles while using NoteExpress as the filter. Searching date: January 1, 2010 to May 1, 2021.

VIP

#1 前交叉韧带损伤 + 前交叉韧带拉伤 + 前交叉韧带撕裂 + 前交叉韧带断裂 + 前十字韧带损伤 + 前十字韧带拉伤 + 前十字韧带撕裂 + 前十字韧带断裂

#2 拉赫曼试验 + lachman 试验 + 轴移试验 + 前抽屉试验

#3 诊断

#4 #1 与 #2 与 #3

Note:We searched 6 articles while using NoteExpress as the filter. Searching date: January 1, 2010 to May 1, 2021.

CBM

("前交叉韧带损伤"[常用字段:智能] OR "前交叉韧带拉伤"[常用字段:智能] OR "前交叉韧带撕裂"[常用字段:智能] OR "前交叉韧带断裂"[常用字段:智能] OR "前十字韧带损伤"[常用字段:智能] OR "前十字韧带拉伤"[常用字段:智能] OR "前十字韧带撕裂"[常用字段:智能] OR "前十字韧带断裂"[常用字段:智能]) AND ("拉赫曼试验"[常用字段:智能] OR "lanchman 试验"[常用字段:智能] OR "轴移试验"[常用字段:智能] OR "前抽屉试验"[常用字段:智能]) AND ("诊断"[常用字段:智能])

Note:We searched 275 articles while using NoteExpress as the filter. Searching date: January 1, 2010 to May 1, 2021.

Chinese Clinical Trial Registry

注册题目：前交叉韧

公开试验结果文件：不限/All

研究类型：诊断试验/Diagnostic test

Note:We searched 1 study. Searching date: January 1, 2010 to May 1, 2021.

Clinical Trials. gov

Study type:All Studies

Study Results:All Studies

Intervention/Treatment:lachman test OR pivot shift test OR anterior drawer test

Note:We searched 3 studies. Searching date: January 1, 2010 to May 1, 2021.

Supplemental Table 1 Details of quality assessment by the QUADAS-2 tool.

references	5	6	7	8	9	10	11	12	13	14	15	16	17	18	19	20	21	22
Was a consecutive or random sample of patients enrolled?	√	√	√	√	√	√	√	√	√	?	×	√	√	√	√	√	√	√
Was a case-control design avoided?	√	√	×	√	×	√	√	√	√	√	√	√	√	√	×	√	×	√
Did the study avoid inappropriate exclusions?	√	√	√	√	√	√	√	√	√	?	√	√	√	√	√	√	√	√
Are there concerns that the included patients and setting do not match the review question?	L	L	L	L	L	L	L	L	L	L	L	L	L	L	L	L	L	L
Were the index test results interpreted without knowledge of the results of the reference standard?	?	√	?	√	√	√	√	?	?	×	√	√	?	√	√	√	?	√
If a threshold was used, was it pre-specified?	×	×	√	×	√	×	×	×	√	×	×	×	×	×	×	√	√	×
Are there concerns that the index test, its conduct, or interpretation differ from the review question?	U	U	U	L	U	L	L	L	L	L	L	L	L	L	L	L	L	L
Is the reference standards likely to correctly classify the target condition?	√	√	√	√	×	√	×	√	√	×	×	√	√	√	√	√	√	√
Were the reference standard results interpreted without knowledge of the results of the index tests?	√	√	√	√	?	√	√	?	√	?	?	?	√	?	√	√	√	√
Are there concerns that the target condition as defined by the reference standard does not match the question?	U	L	L	L	L	L	L	U	L	L	U	L	L	L	L	L	L	L
Was there an appropriate interval between index test and reference standard?	√	?	?	?	√	?	?	?	?	√	√	?	?	√	√	√	?	?
Did all patients receive the same reference standard?	√	×	√	×	×	×	√	√	√	√	×	√	√	√	√	√	√	√
Were all patients included in the analysis?	√	×	√	×	×	×	×	√	√	√	√	×	√	√	×	×	√	√

Notes:√=yes; ×=no; ?=unclear;L=low risk;U=unclear risk;H=high risk

Supplemental Table 2 Characteristics of the LT.

ID	Study name	Reference standard	TP	FP	FN	TN
1	Han et al 2010	Arthroscopic visualization	39	9	13	19
2	Han et al 2010	Arthroscopic visualization	49	6	3	22
3	Han et al 2010	Arthroscopic visualization	40	9	12	19
4	Mulligan et al 2011	Arthroscopic visualization and application of clinical cluster of findings	16	1	7	28
5	Mulligan et al 2011	Arthroscopic visualization and application of clinical cluster of findings	18	3	5	26
6	Mulligan et al 2015	Arthroscopic visualization and application of clinical cluster of findings	14	0	3	28
7	Tanaka et al 2017	Clinical findings, magnetic resonance imaging, and arthroscopic observation	20	1	4	23
8	Mulligan et al 2017	Arthroscopic visualization and application of clinical cluster of findings	20	4	4	32
9	Mulligan et al 2017	Arthroscopic visualization and application of clinical cluster of findings	16	1	8	35
10	Massey et al 2017	Magnetic resonance imaging	63	3	8	17
11	Wu et al 2017	Arthroscopic visualization	84	1	6	9
12	Cai et al 2017	Arthroscopic visualization	130	7	57	16
13	Kıyak et al 2018	Magnetic resonance imaging	14	4	24	20
14	Kıyak et al 2018	Magnetic resonance imaging	17	2	21	22
15	Kıyak et al 2018	Magnetic resonance imaging	22	1	16	23
16	Kıyak et al 2018	Magnetic resonance imaging	26	2	12	22
17	Décary et al 2018	Magnetic resonance imaging	35	5	8	231
18	Lichtenberg et al 2018	Arthroscopic Surgery	41	4	6	42
19	Krakowski et al 2019	Knee arthroscopy findings	27	5	5	59
20	Gürpınar et al 2019	Arthroscopic visualization	50	6	12	10
21	Gürpınar et al 2019	Arthroscopic visualization	52	5	10	11
22	Blanke et al 2020	Arthroscopic visualization	74	17	26	83
23	Feng 2020	Arthroscopic visualization	19	8	11	22
24	Feng 2020	Arthroscopic visualization	16	1	14	29
25	Zhao et al 2021	Arthroscopic visualization	117	27	31	225

Supplemental Table 3 Characteristics of the ADT.

ID	Study name	Reference standard	TP	FP	FN	TN
1	Han et al 2010	Arthroscopic visualization and rthroscopic Surgery	24	11	28	17
2	Zhao et al 2013	Arthroscopic visualization	34	3	2	37
3	Massey et al 2017	Magnetic resonance imaging	58	4	13	16
4	Wu et al 2017	Arthroscopic visualization	73	1	17	9
5	Cai et al 2017	Arthroscopic visualization	115	9	72	14
6	Kıyak et al 2018	Magnetic resonance imaging	10	6	28	18
7	Kıyak et al 2018	Magnetic resonance imaging	14	4	24	20
8	Kıyak et al 2018	Magnetic resonance imaging	12	4	26	20
9	Kıyak et al 2018	Magnetic resonance imaging	18	3	20	21
10	Lichtenberg et al 2018	Arthroscopic Surgery	32	3	13	43
11	Krakowski et al 2019	Knee arthroscopy findings	22	4	10	60
12	Gürpınar et al 2019	Arthroscopic visualization	48	5	14	11
13	Gürpınar et al 2019	Arthroscopic visualization	49	4	13	12
14	Zhao et al 2021	Arthroscopic surgery	95	17	53	235

Supplemental Table 4 Characteristics of the PST.

ID	Study name	Reference standard	TP	FP	FN	TN
1	Han et al 2010	Arthroscopic visualization and rthroscopic Surgery	27	1	25	27
2	Massey et al 2017	Magnetic resonance imaging	44	1	23	15
3	Wu et al 2017	Arthroscopic visualization	84	0	6	10
4	Kıyak et al 2018	Magnetic resonance imaging	8	2	30	22
5	Kıyak et al 2018	Magnetic resonance imaging	12	2	26	22
6	Kıyak et al 2018	Magnetic resonance imaging	16	1	22	23
7	Kıyak et al 2018	Magnetic resonance imaging	21	2	17	22
8	Décary et al 2018	magnetic resonance imaging	33	5	10	231
9	Lichtenberg et al 2018	Arthroscopic Surgery	18	1	18	44
10	Krakowski et al 2019	Knee arthroscopy findings	14	1	18	63
11	Gürpınar et al 2019	Arthroscopic visualization	32	1	30	15
12	Gürpınar et al 2019	Arthroscopic visualization	35	1	27	15
13	Blanke et al 2020	Arthroscopic visualization	46	4	54	96
14	Murgier et al 2020	Ultrasonography	56	2	8	64
15	Zhao et al 2021	Arthroscopic surgery	91	9	57	243

Supplemental Table 5 Characteristics of the LST.

ID	Study name	Reference standard	TP	FP	FN	TN
1	Mulligan et al 2017	Arthroscopic visualization and application of clinical cluster of findings	9	10	15	26
2	Massey et al 2017	Magnetic resonance imaging	59	4	12	16
3	Wu et al 2017	Arthroscopic visualization	85	0	5	10
4	Lichtenberg et al 2018	Arthroscopic Surgery	16	0	25	46
5	Krakowski et al 2019	Knee arthroscopy findings	20	1	12	63
6	Gürpınar et al 2019	Magnetic resonance imaging	57	1	5	15
7	Gürpınar et al 2019	Magnetic resonance imaging	57	1	5	15
